# Role of tranexamic acid in reducing peri-operative blood loss in open spine surgeries

**DOI:** 10.1007/s12306-024-00826-0

**Published:** 2024-05-31

**Authors:** Madhava Pai Kanhangad, V. Ramachandra Theja, Shyamasunder N. Bhat

**Affiliations:** Department of Orthopaedics, Kasturba Medical College, Manipal, Manipal Academy of Higher Education, Manipal, Karnataka 576104 India

**Keywords:** Tranexamic acid, Spine surgery, Blood loss, Blood transfusion, Surgical drain

## Abstract

Spine surgeries are associated with significant blood loss due to the extensive soft tissue dissection, bony decompression, and prolonged surgical time. Excessive bleeding may require blood transfusions and thereby increase the risk of adverse transfusion reactions. Therefore, minimizing peri-operative bleeding is important for spine surgeons to reduce post-operative morbidity. Tranexamic acid (TXA) is a synthetic anti-fibrinolytic drug, which helps in reducing perioperative blood loss in major surgeries. The evidence on the efficacy of this agent in all manner of spine surgeries is not sufficient. Hence this study was conducted to determine the efficacy of TXA on perioperative blood loss in major spinal surgeries. In a prospective study, two groups of patients with similar surgical profiles who were posted for all manner of open spine surgeries were included. One group received one gram of intravenous TXA while the others did not. Intra- and post-operative assessments included noting levels of surgery, duration of surgery, assessment of blood loss, intra- and/or post- operative blood transfusion, and blood collected in surgical drain at the end of 24 h. The intra-operative blood loss, frequency of intra-operative blood transfusion, post-operative Hemoglobin drop, and surgical drain output were found to be significantly lower in patients who received TXA. In spine surgeries, TXA was found to be effective in reducing intra-operative blood loss, need for intra-operative blood transfusion and post-operative Hb drop. Also, TXA had reduced surgical drain output significantly between the two groups.

## Introduction

Spine surgeries are complex procedures which are sometimes associated with a significant amount of bleeding due to the soft tissue dissection, decompression, and prolonged surgical time [1–3]. The bleeding associated with the surgical procedure may require blood transfusion, which carries with it the risk of adverse transfusion-related reactions. Perioperative bleeding during open spine surgeries may also result in formation of epidural hematoma, which could lead to compression of roots or the spinal cord causing neurological deficits in patients. An increase in healthcare-related costs for procuring blood and blood products, and prolonged hospital stay in those receiving blood transfusions has been noted [4–7]. Therefore, minimizing perioperative bleeding is important to reduce post-operative morbidity. Various techniques to reduce perioperative blood loss have been described and thereby decrease the requirement of a perioperative blood transfusion. Some of the commonly followed methods are, controlled hypotension during the surgical procedure, autologous blood transfusion by salvaging blood intraoperatively, meticulous surgical techniques with achievement of adequate hemostasis during all steps of surgery and administration of various medications ranging from synthetic formulations to recombinant molecules[8–10]. A simple, cost-effective, readily available agent is Tranexamic acid (TXA) [11]. This synthetic anti-fibrinolytic compound which is a derivative of the lysine-binding site of the plasminogen, acts by inhibiting the plasminogen from binding to the fibrin molecule and suppressing fibrinolysis, thereby decreasing the perioperative blood loss in major surgical procedures [11]. This drug is widely used for joint replacement surgeries and has positive results as illustrated by various studies [12]. However, the evidence related to the efficacy of this drug in all manner of open spine surgeries is not sufficient [13]. This study aimed to determine the effectiveness of a single preoperative bolus dose of TXA at reducing perioperative blood loss in all manner of open spine surgeries.

## Materials and methods

Assuming a standard difference of 200 mL of blood loss between patients who receive TXA compared to those who do not, a power of 80% and a 5% level of significance, the sample size was estimated to be 44, in each group and this was rounded off to 50 patients in each group [14]. In a prospective observational study, one hundred and twenty-seven American Society of Anesthesiologists class I and II patients above the age of 18 years undergoing all manner of open spine surgeries at a tertiary referral between June 2021 to June 2022 were included in the study. Patients who had undergone unilateral single level fenestration discectomies, those with blood dyscrasias, coagulation profile abnormalities, and renal disorders, patients in whom duration of surgery was more than 6 h, were excluded. Patients on medication which could interfere with tranexamic acid such as aspirin, clopidogrel, dabigatran, heparin, enoxaparin, fondaparinux were also excluded. The patients who declined to participate were also excluded. Thus one hundred patients, who provided written informed consent, formed the basis of the study. The study was conducted after obtaining institutional ethical committee.

Pre-operative assessment included recording of the patient’s age, gender, body mass index, presence of comorbidities, preoperative Hemoglobin, platelet count, Prothrombin Time/international normalized ratio (PT/INR) and Activated Partial Thromboplastin Clotting Time (APTT), Serum urea, and serum creatinine values.

The patients were divided into two groups using block randomization. Those in Group A received one gram of intravenous TXA thirty minutes prior to skin incision; whereas, those in Group B did not receive this drug. All surgeries were performed by one of three trained spine surgeons and all surgeries were done under general anesthesia. No other strategies to minimize blood loss like, autologous transfusion and levels of decompression, duration of surgery, assessment of blood loss (mops, gauze count, suction apparatus), need for any intra-operative blood transfusion were noted. Intra-operative blood transfusion was decided based on the standard guidelines laid down by Association of Anaesthetists of Great Britain and Ireland [15]. Intra-operative blood loss assessment was done by a single observer for all cases. Total volume of fluid in suction apparatus (*a*), normal saline (*b*) and other fluids used in surgery (*c*) was noted. Therefore, the blood loss in suction apparatus was determined by [*a* − (*b* + *c*)]. Total weight of mops and gauzes before surgery (*d*), and total weight of mops and gauzes after surgery (*e*) was noted. Blood loss in mops and gauzes was determined by the formula (*e* − *d*), assuming a density of 1 g/mL. Blood loss in surgical drain (*f*) was measured 24 h after surgery. The total blood loss was calculated using the formula.

$$\left[ {a\, - \,\left( {b\, + \,c} \right)} \right]\, + \,\left[ {e\, - \,d} \right]\, + \,f$$

Post-operative assessment includes repeating the following parameters Hemoglobin, PT/INR, APTT after 24 h of surgery. All the surgical drains had negative suction pressure till their removal. The blood collected in surgical drain was measured using a standard measuring jar and documented. Post-operative blood transfusion, if any, was done based on the guidelines laid down by Association of Anaesthetists of Great Britain and Ireland [15].

Postoperatively patients were assessed clinically for any symptoms of deep vein thrombosis.

Continuous variables like age, hemoglobin were expressed as mean ± SD; while, quantitative variables such as sex, frequency of blood transfusion was expressed as percentage. The student t-test was used to compare means. The frequencies between the two groups were compared using the chi squared test. Statistical Package for Social Sciences for Windows version 20 was used to perform all analysis.

## Results

Demographic data, and preoperative blood investigations are shown in Table 1, and were found to be comparable between the two groups. The frequency of indication for surgery in the two groups is shown in Table 2 and was found to be comparable. The mean number of levels of instrumentation was 2.5 levels and 2.7 levels in group A and B respectively. The mean duration of surgery was also comparable between the two groups. The chi squared test showed patients in group A had a lower frequency of intra-operative blood transfusion as compared to those in group B (*p* = 0.028). However, there was no statistically significant difference in the frequency of post-operative transfusion between the two groups (*p* = 0.11). The details of intra- and post-operative blood loss are shown in Table 2. The intra-operative blood loss, intra-operative frequency of blood transfusion, surgical drain at 24 h, and total blood loss was found to be significantly lower in group A compared to group B. However there was no statistically significant difference in frequency of post-operative transfusion. None of the patients had an anaphylactic reaction to TXA. None of the patients developed deep vein thrombosis at discharge (Table 3).

**Table 1:** Table showing demographic, surgical data and preoperative blood investigations of the two groups

	Group A (*n* = 50)	Group B (*n* = 50)	*p* Value
Age	47.9 ± 15.4	48.8 ± 16.8	0.795*
Male:Female	74%/26%	58%/42%	0.091**
Pre-operative Hemoglobin (g/dL)	12.7 ± 1.9	12.5 ± 2	0.628*
Pre-operative Platelet count	38 ± 5.1	37.7 ± 5.4	0.803*
Pre-operative PT	11.2 ± 0.8	11.2 ± 0.9	0.733*
Pre-operative aPTT	27.3 ± 3	27.9 ± 3.2	0.331*
Cervical Surgeries	2	3	0.868**
Thoracic Surgeries	18	20	
Lumbar surgeries	23	22	
Surgical duration (mins)	158.5 ± 23.1	162.8 ± 25.7	0.38*

^*^Independent sample t test, **Chi-squared test

**Table 2:** Table showing the frequency of indication of surgery between the two groups

	Group A (*n* = 50)	Group B (*n* = 50)	*p* Value
Spinal injury	14	15	0.84*
Spondylodiscitis	13	11	
Lumbar canal stenosis	9	8	
Pathological lesion	4	7	
Spondylolisthesis	5	7	
Myelopathy	3	1	
Implant removal	2	1	

^*^Chi-squared test

**Table 3:** Table showing intra-operative blood loss, frequency of transfusions, post-operative hemoglobin, surgical drain at 24 h and total blood loss (study variables) and their comparison

	Group A (*n* = 50)	Group B (*n* = 50)	*p* Value
Intra-operative blood loss (mL)	698.90 ± 230.3	1173.80 ± 302.8	< 0.001*
Intra-operative transfusion frequency	40%	62%	0.028**
Post-operative Hemoglobin (g/dL)	11.82 ± 1.7	11.06 ± 1.9	0.038*
Surgical Drain at 24h (mL)	152.10 ± 151.8	216.50 ± 68.8	< 0.001*
Post-operative transfusion frequency	6%	16%	0.110**
Total Blood loss (mL)	851 ± 324	1390.30 ± 327.9	< 0.001 *

^*^Independent sample t test, **Chi-squared test

## Discussion

Blood loss is inevitable during open spine surgery, and when it exceeds physiological compensatory mechanisms, it warrants treatment by blood transfusions. Various methods have been used to minimize intra-operative blood loss, to avoid the higher health care costs and prolonged hospitalization associated with blood transfusions. In our study, we attempted to determine the efficacy of preoperative TXA in reducing perioperative blood loss. Variables like age, gender, pre-operative hemoglobin, pre-operative coagulation profile, type of the surgical procedure, levels of decompression, duration of the surgical procedure, post-operative coagulation profile were comparable between the two groups, thereby minimizing confounding factors. The mean intra-operative blood loss was found to be 40.49% lesser in group A compared to group B in our study.

Wong et al. [16] found that there was 24.81% reduction in the intra-operative blood loss between the study and control groups. This study only included elective thoracic and lumbar instrumented spine fusions. In their study, patients with anemia were excluded and all the patients with a hemoglobin value of less than 13 g/dL received erythropoietin supplements pre-operatively. None of the patients in our study were optimized to such an extent and our study included both elective and emergency cases and patients with anemia were not excluded. Anemia is a risk factor for excessive bleeding during a surgical procedure, which could explain the greater difference in blood loss noted in this study. Elwatidy et al. [17] found a 49% reduction in the intra-operative blood loss between the study and control groups. However, in their study, a loading dose of two grams of intravenous TXA was given followed by a continuous infusion, in the study group. In our study, only a single dose of one gram intravenous Tranexamic acid was given thirty minutes before skin incision. A study by Wang et al. [18] concluded that there was no statistically significant reduction in the intra-operative blood loss between the study and control groups but the total blood loss which is a combination of intra-operative and post-operative blood loss was statistically significant. This observation could be because of a small sample size and only posterior lumbar interbody fusion surgeries were considered.

There was 22% lower frequency of intra-operative blood transfusion in group A compared to group B in our study. Other studies had the frequency range from 4 to 80% [9–11]. This could be because other studies combined other strategies like intra-operative cell saver and autologous blood transfusion; while, other studies used an infusion of TXA. To avoid confounding factors, our study did not use any other strategy to minimize intra-operative blood loss.

The drop in post-operative hemoglobin in group A was 17.8% lesser in comparison with group B in our study. Wong et al. noted that there was 5.3% reduction in the post-operative hemoglobin drop between the study and control groups which was significant but lesser than that found in our study. This difference was lesser compared to our study because autologous transfusion was done intraoperatively if more than 800 ml of blood loss was present, in the study by Wong et al. [16]. Elwatidy et al. concluded that there was 8.39% reduction in the post-operative hemoglobin drop between the study and control groups (*p*-0.006) [16]. In our study all post-operative hemoglobin values were obtained 24 h after surgery.

The blood collected in surgical drain was 29.62% lesser in group A compared to group B in our study, similar to the findings of other studies [16, 18]. However, Elwatidy et al. a 54.51% reduction in the surgical drain output between the study and control groups [17]. This is probably due to the higher bolus dose of Tranexamic acid and maintenance of infusion for 5 h post-surgery.

There was no statistically significant difference between the two groups with respect to frequency of post-operative blood transfusion in our study. The study by Elwatidy et al. published in 2008 concluded that there was 66% reduction in the frequency of post-operative blood transfusion between the study and the control groups. This is probably due to the higher bolus dose of intravenous TXA and maintenance of infusion of TXA for 5 h post-surgery which had decreased the post-operative surgical drain output by 54.51% between the two groups [17].

Coagulation profile was done pre-operatively and postoperatively for all patients and there was no statistically significant derangement between group A and group B which proves that TXA does not alter patient’s blood coagulation parameters. None of the patients in our study developed any thromboembolic complications like deep vein thrombosis. A meta-analysis by Li et al. [11] published in 2013 concluded that there were no post-operative complications especially thromboembolic events in the patients who received TXA preoperatively.

The strength of our study is the inclusion of all manner of surgeries, including cervical spine stabilization and spinal implant removals. All blood loss estimations were done by a single trained independent observer, to minimize observer bias. The drawbacks of the study are that a single fixed dose of TXA was given as opposed to an infusion, which may affect its concentration in blood in different individuals. The surgeons were not blinded to administration of TXA and therefore may result in some confounding factors.

This study demonstrates that TXA is a safe and effective pharmacological agent in reducing perioperative blood loss. Further studies could help optimize the dose and dosage in patients undergoing spine surgery.
